# Estimation of porcine pancreas optical properties in the 600–1100 nm wavelength range for light-based therapies

**DOI:** 10.1038/s41598-022-18277-7

**Published:** 2022-08-22

**Authors:** Pranav Lanka, Leonardo Bianchi, Andrea Farina, Martina De Landro, Antonio Pifferi, Paola Saccomandi

**Affiliations:** 1 Department of Physics, Politecnico di Milano, 20133 Milan, Italy; 2 Department of Mechanical Engineering, Politecnico di Milano, 20156 Milan, Italy; 3 Institute of Photonics and Nanotechnologies, National Research Council, 20133 Milan, Italy

**Keywords:** Biophotonics, Biomedical engineering

## Abstract

This work reports the optical properties of porcine pancreatic tissue in the broad wavelength range of 600–1100 nm. Absorption and reduced scattering coefficients (*µ*_a_ and *µ*_s_′) of the ex vivo pancreas were obtained by means of Time-domain Diffuse Optical Spectroscopy. We have investigated different experimental conditions—including compression, repositioning, spatial sampling, temporal stability—the effect of the freezing procedure (fresh vs frozen-thawed pancreas), and finally inter-sample variability. Good repeatability under different experimental conditions was obtained (median coefficient of variation less than 8% and ~ 16% for *µ*_a_ and *µ*_s_′, respectively). Freezing–thawing the samples caused an irreversible threefold reduction of *µ*_s_′ and no effect on *µ*_a_. The absorption and reduced scattering spectra averaged over different samples were in the range of 0.12–0.74 cm^−1^ and 12–21 cm^−1^ with an inter-sample variation of ~ 10% and ~ 40% for *µ*_a_ and *µ*_s_′, respectively. The calculated effective transport coefficient (*µ*_*eff*_) for fresh pancreatic tissue shows that regions between 800–900 nm and 1050–1100 nm are similar and offer the lowest tissue attenuation in the considered range (i.e., *µ*_*eff*_ ranging from 2.4 to 2.7 cm^−1^). These data, describing specific light-pancreas interactions in the therapeutic optical window for the first time, provide pivotal information for planning of light-based thermotherapies (e.g., laser ablation) and instruction of light transport models for biophotonic applications involving this organ.

## Introduction

Pancreatic cancer is an aggressive malignancy accounting for more than 466,000 deaths and 495,770 new cases in 2020 worldwide^1^. In the USA, pancreatic cancer currently represents the fourth leading cause of cancer death, and, since the number of deaths due to this lethal disease is rapidly increasing, it is estimated to become the second leading cause of tumor-related death by 2030^2^.

The possible treatments currently available typically include surgery, radiation therapy, and chemotherapy. However, most systemic therapies have not succeeded in ameliorating patients’ prognosis, showing limited clinical benefits^3^. At present, surgical resection, i.e., pancreatectomy, represents the only widely accepted treatment option with the potential to increase long-term survival. However, only 20% of the patients are appropriate surgical candidates at the time of the diagnosis. Moreover, the complexity and invasiveness as well as the strict dependence of the overall result on the operator’s ability and experience restrain the applicability of this treatment approach^4^. Therefore, novel therapeutic strategies are emerging^5–7^. Among these, thermal ablation procedures demonstrated encouraging results^8^: they aim at reducing cancer tumor volume, to achieve better local disease control, with the final intent of improving survival and quality of life^9^. Laser ablation (LA) technique, in particular, is a promising light-based ablative procedure that relies on a tissue temperature increase due to the photothermal conversion of laser radiation into heat. Malignant tissues, exposed to laser light, are therefore subjected to a localized and cytotoxic temperature rise while surrounding healthy structures are preserved from thermal damage^10^.

The rate of adverse events for LA is lower than with other thermal techniques^11,12^, and, among all thermal treatment modalities, LA is unique in enabling the use of a finer needle (i.e., diameter < 1 mm^10^). Indeed, LA represents an attractive option for the treatment of focal lesions in high-risk locations, difficult-to-reach sites, or multiple nodules that differ in size. These advantages encourage the utilization of LA for the treatment of organs with delicate anatomical positions, such as the pancreas, as witnessed by recent studies on the endoscopic ultrasound-guided LA in locally advanced, unresectable pancreatic adenocarcinoma^13^. However, the clinical application of LA for pancreatic tissue treatment is still hampered by the limited knowledge of the pancreas physical properties^14,15^ and the necessity to optimize the procedural setting parameters^4^.

The physical mechanism underlying LA is dictated by the laser-tissue interaction, i.e., the penetration of the light into the biological media and the subsequent energy deposition due to the transfer of photon energy to the tissue. Particularly, when light interacts with tissue, it undergoes scattering and absorption phenomena. While the optical scattering regulates the light propagation direction and can be ascribed to the interaction of the photons with cellular and subcellular structures, light absorption entails a localized temperature increase due to the presence of specific tissue chromophores^16^. Penetration depth is the measure of how deeply light can penetrate the medium and depends on the tissue scattering and absorption properties, whose combined effect leads to the effective transport coefficient, which ultimately rules light attenuation for a highly scattering medium^17^.

Endogenous chromophores and tissue constituents are characterized by a wavelength-dependent optical behavior, hence the investigation of the organ-specific optical properties as a function of wavelength is pivotal for targeted therapeutic and diagnostic applications^18,19^. For LA of biological soft tissues, a good balance between the desired light penetration depth and absorption is needed and it is typically shown within the 650–1300 nm optical window, i.e., therapeutic window^20^. Therefore, the analysis of the optical behavior of the pancreatic tissue in a range comprised within this interval is necessary to characterize the landscape of optical properties associated with this organ, for manifold purposes. Firstly, information on the optical characteristics may allow one to optimize the irradiation procedure by selecting the adequate laser specifications, e.g., laser wavelength, to obtain the required light penetration and absorption. Moreover, the attained tissue-specific optical coefficients are useful for implementing accurate predictive tools of the light-to-heat conversion for treatment planning^21,22^. These simulation-based models are particularly advantageous for estimating the light propagation and temperature distribution due to the photothermal effect. Accurate modeling can therefore support clinicians towards new treatment paradigms and the design of procedures specifically tailored to pancreatic tissue^23^. This is also crucial to determine the optimal procedural settings and the best LA strategy with the aim to improve the final clinical outcome.

However, the literature lacks satisfactory information on the optical response of the pancreas, thus limiting the complete understanding of the light-to-heat conversion for therapeutic purposes. In some cases, the shortage of optical properties for pancreatic tissue has been bridged by using properties of more characterized tissue (e.g., liver) for simulation purposes, thus impacting the accuracy of the results^24^.

In this study, we propose the optical characterization of ex vivo porcine pancreatic tissue over a broad wavelength range of 600–1100 nm. Absorption and reduced scattering coefficients (i.e., *µ*_a_ and *µ′*_s_) of the tissue were attained using Time-domain Diffuse Optical Spectroscopy (TD-DOS) which permits the natural disentanglement of these two coefficients^25^. Porcine tissues showcase strong similarities to human tissue in chromophore and constituent concentrations, making them ideal choices for use as biological phantoms to model human heterogeneity and complex structure^26–28^. Several experimental conditions, such as the intra-sample variation, the compression testing, the repositioning error, along with the measurement stability, have been investigated. Furthermore, the influence of the tissue storage method on the pancreas optical properties has been evaluated by analyzing freshly excised and frozen samples. This information would be particularly useful for the refinement of protocols and laboratory practices for the maximum reliability of experiments involving pancreatic tissue, as well as for instructing numerical simulations.

## Results

### Analysis of the influence of measurement conditions

**Figure 1:**
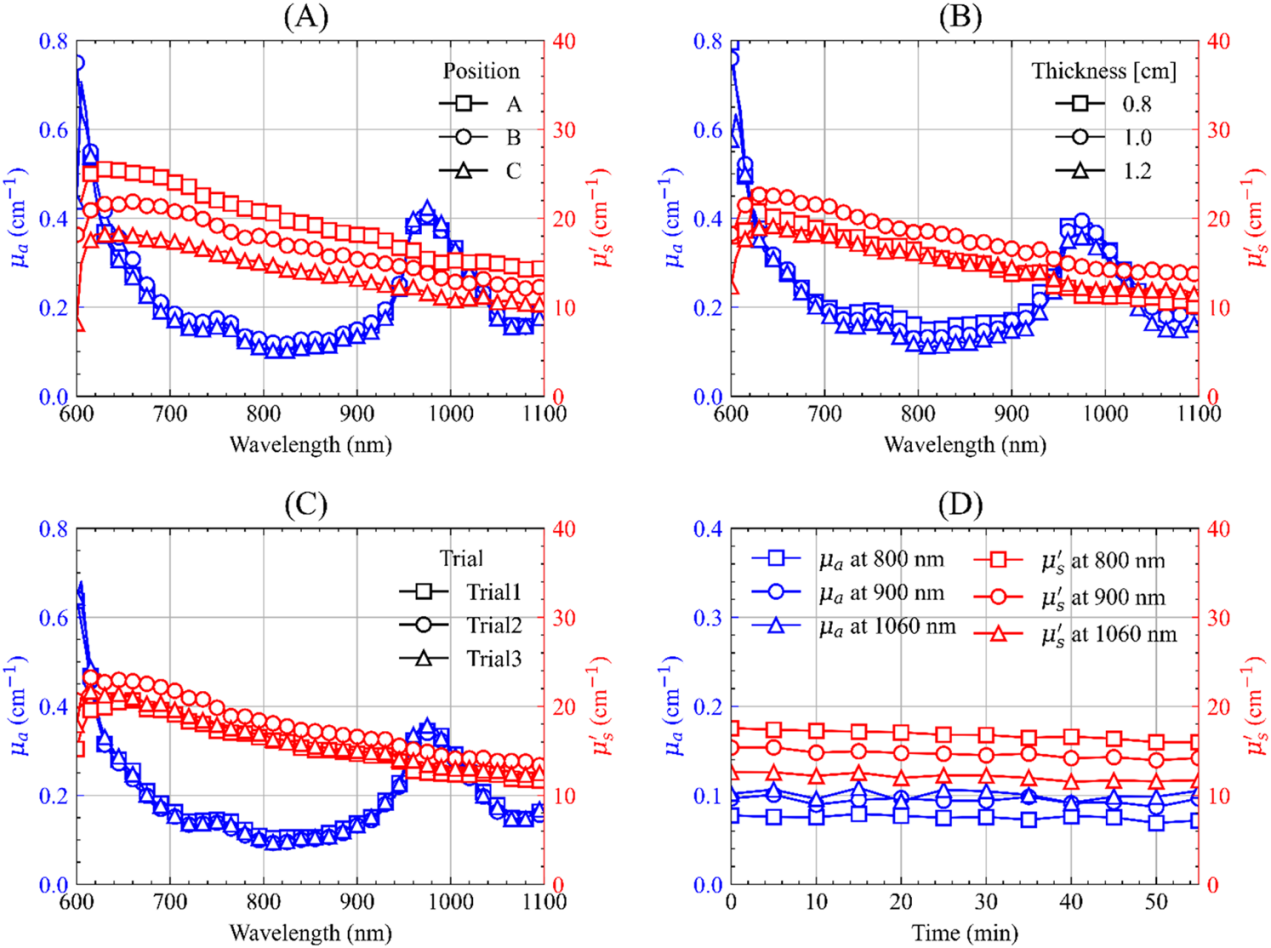
Influence of different types of measurement conditions on the optical properties of fresh porcine pancreatic tissues: (**A**) intra-sample variation, (**B**) sample compression, (**C**) sample repositioning, (**D**) stability in the recovered optical properties over 1 h at 800, 900 and 1060 nm. Each subplot represents the results attained in a single organ.

The results of the tests concerning the intra-sample variation, and the influence of sample compression, repositioning, and the stability of the measurement over extended periods can be seen in the four subplots of Fig. 1. In general, all these tests do not highlight any major changes in the absorption coefficient either qualitatively or quantitatively. The median over wavelength coefficient of variation (defined as the stdev/mean) for intra-sample variation, compression and repositioning tests in *µ*_*a*_ are 5%, 8% and 3%, respectively. For the reduced scattering coefficient spectra these values correspond to 16%, 10% and 7% showing a noticeable influence of the intra-sample variation of the recovered reduced scattering spectrum. The tissue in the fresh configuration also presents good temporal stability with a variation of about 0.003 cm^−1^/h in *µ*_*a*_ and 1.2 cm^−1^/h in *µ′*_*s*_.

### Optical properties of fresh pancreas

**Figure 2:**
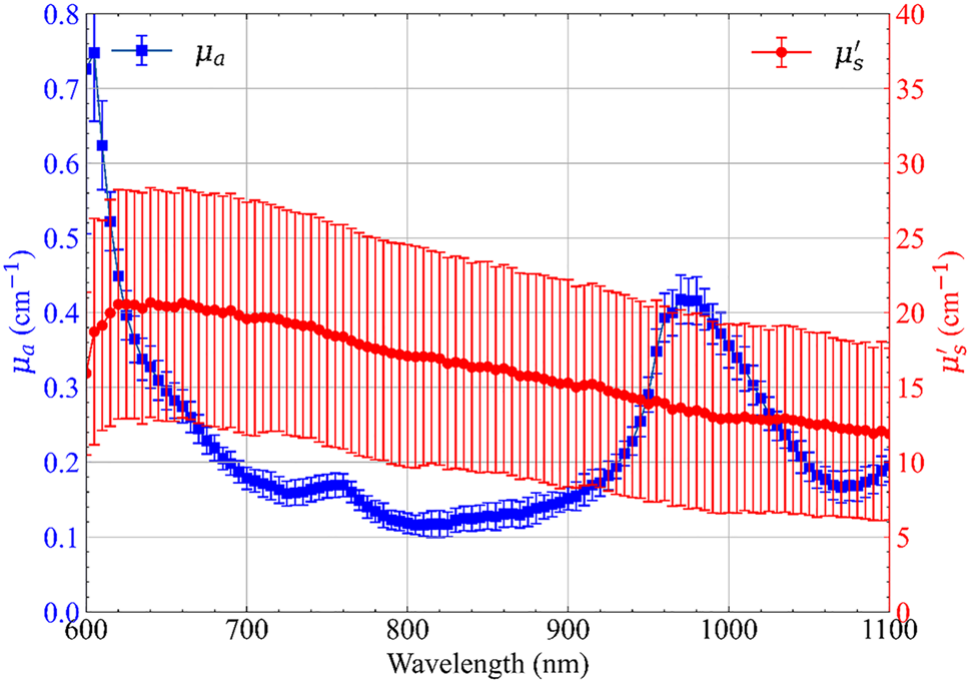
Average absorption (*µ*_*a*_) and reduced scattering spectra (*µ′*_*s*_) of fresh ex vivo porcine pancreatic tissue. Measurements were performed over 5 samples at 3 different positions (A–C) on each sample. The error bars represent the standard deviation over the 15 measurements.

The average spectra of *µ*_*a*_ (blue squares) and *µ′*_*s*_ (red circles) of the freshly procured ex vivo porcine pancreas considered for this study are presented in Fig. 2. The figure summarizes the results of a total of 15 measurements. The markers represent the average values, and the error bars represent the standard deviation over these 15 measurements.

The absorption spectrum is defined predominantly by three spectral features namely, (1) a broad peak at 980 nm, (2) a relatively smaller and subtler peak at 760 nm, (3) a strong decreasing absorption tail in the red region of the spectrum (below 650 nm). The main peak is related to water (a vibrational overtone of the O–H bond stretching), while the second peak and the decreasing tail to the high blood content of the pancreatic tissue. The maximum value of *µ*_a_ is 0.74 cm^−1^ @ 605 nm, while its minimum value is 0.12 cm^−1^ @ 805 nm. The relatively small error bars on the absorption spectrum (median over wavelength coefficient of variation amongst the 15 measurements ~ 8%) indicate that the absorption features of the tissue across samples are not only consistent in spectral trends but in absolute value as well.

The reduced scattering spectrum exhibits the well-known decreasing trend with wavelength with no particular peaks or other spectral features^29^. In fact, this downward trend of the reduced scattering spectrum has been studied extensively and is approximated by an empirical power-law derived from the Mie theory of the form^30^:

1 $$\mu ^{\prime}_{s} (\lambda ) = a\left( {\frac{\lambda }{{\lambda_{0} }}} \right)^{ - b}$$

where *a* and *b* are the scatter amplitude and scatter power which are related to the density and size of the scattering centers. Considering the mean values calculated over the 5 samples and at 3 different positions, the maximum value of *µ′*_s_ is 21 cm^−1^, @ 640 nm, whereas its minimum is 12 cm^−1^, @ 1100 nm. Here we observe relatively larger error bars (median over wavelength coefficient of variation amongst the 15 measurements ~ 42%), indicating major variations in absolute values between different samples and different spatial locations. This could just be on account of the variations in the structural composition of the tissue across the different samples considered for the study. Moreover, as depicted in Fig. 1A, the intra-sample variation has a considerable effect on the absolute values of *µ′*_*s*_, which further substantiates the larger error bars observed in the scattering spectrum of Fig. 2.

### Optical properties of frozen-thawed pancreas

**Figure 3:**
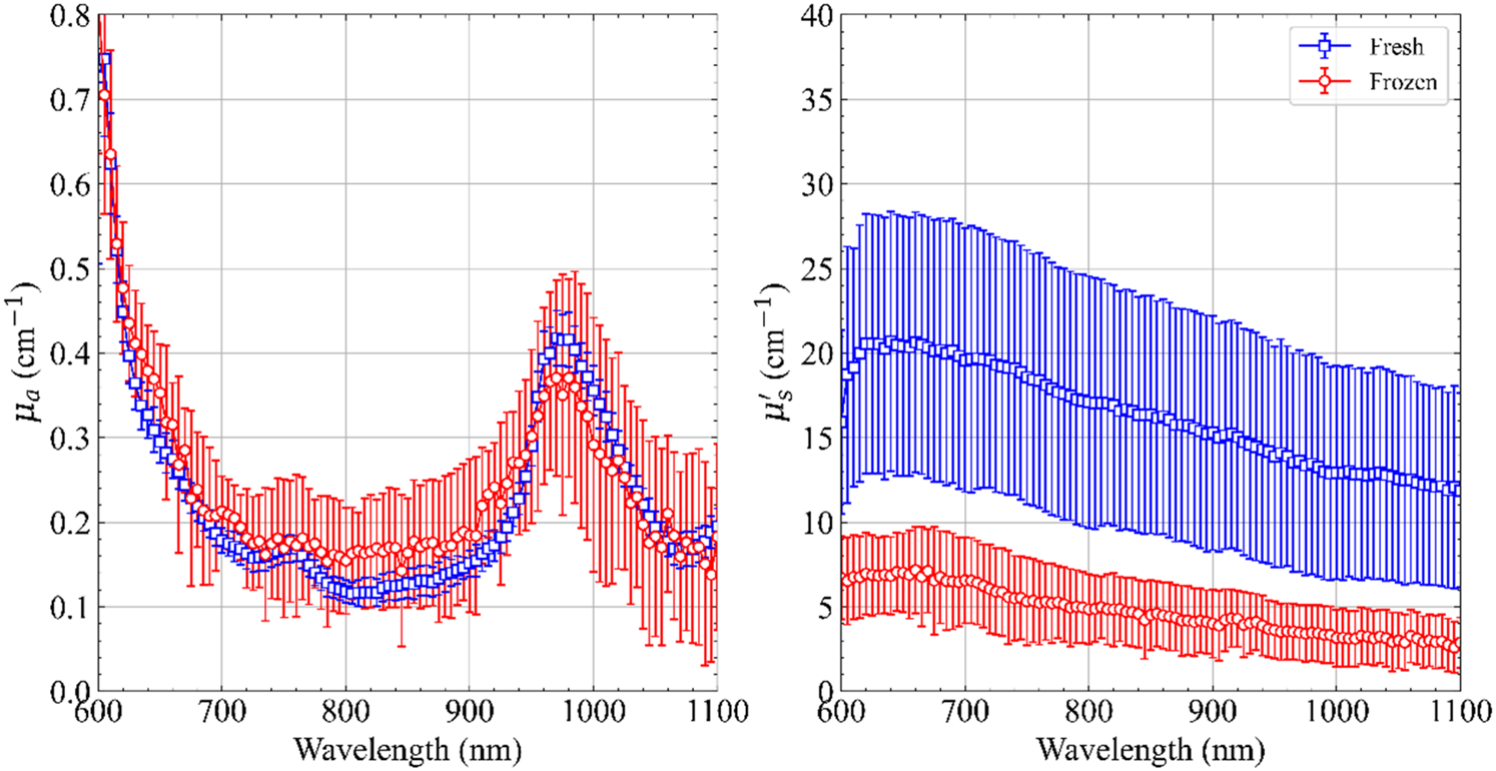
Difference in the average optical properties of the same 5 ex vivo porcine pancreatic samples measured both in the ‘fresh’ and ‘frozen’ state. Each sample is measured at 3 positions (A–C). The error bars represent the standard deviation over the 15 measurements.

In Fig. 3, we compare the optical properties of the same set of 5 tissue samples measured at 3 positions each in (1) fresh (immediately after procuring the tissue from the sacrificed sample) and (2) frozen (after freezing the sample at sub-zero temperatures for 20 h, for preservation purposes, and then defrosting the sample before measurement). Tissue freezing is a convenient method to store and transport tissue samples, and it is general practice to freeze tissue samples that are not immediately utilized for experiments. This test was aimed at understanding if this conservation procedure could be applied for ex vivo pancreatic tissue undergoing LA experiments or other optical studies.

Even in this case, the absorption coefficient spectrum experiences limited changes in both value and spectral trends. However, the reduced scattering spectrum undergoes a significant alteration both in spectral slope and absolute values. This is reflected in the *a* and *b* parameters recovered from the mean values of the two reduced scattering spectra of Fig. 3 and using Eq. (1) (see Table 1). In particular, the *µ*_s_′ spectrum of frozen pancreas exhibits a threefold reduction of average values when compared to the fresh samples.

**Table 1:** Values of scatter amplitude and scatter power attained for fresh pancreas and pancreatic tissue undergoing the freezing procedure.

	Scatter amplitude (a) (cm^−1^)	Scatter power (b)
Fresh	24.1	1.16
Frozen	8.4	1.18

The increased dispersion of data in the absorption spectrum (error bars) can be ascribed to the relatively low scattering coefficient which—combined with the small tissue thickness—challenge the time-resolved techniques in distinguishing the DTOF from the instrument response function.

### Spectral attenuation of pancreatic tissue

Key information needed when treating pancreatic tissue with laser light is the radial distribution of energy from the source, which is related to the effective transport coefficient. From data reported in Fig. 2, we calculated the effective transport coefficient

2 $$\mu_{eff} \left( \lambda \right) = \sqrt {3\mu_{a} \left( \lambda \right)\mu ^{\prime}_{s} \left( \lambda \right)}$$

**Figure 4:**
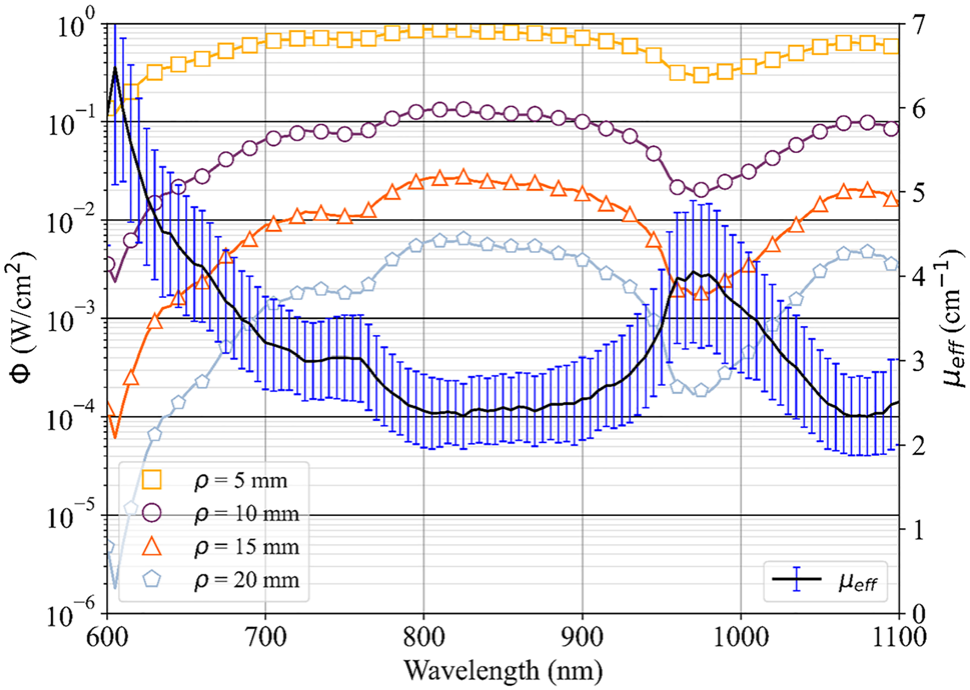
Fluence rate (φ) for different radial distances r from the injection point, left axis, and effective transport coefficient (*µ*_*eff*_*,* expressed as mean values ± standard deviation), right axis, as a function of wavelength.

Being a function of *µ*_*a*_ and *µ’*_*s*_, the coefficient *µ*_*eff*_ governs the light propagation in the tissue when only continuous wave signal is used. Moreover, it can be used to derive the radial dependence of the fluence rate *ϕ*(*λ*, *r*) on the distance *r* from the injection point. Under the Diffusion approximation to the Radiative Transport Equation, and assuming an infinite medium with a point light source (e.g. a fiber tip inserted into the tissue for laser thermal treatment), *ϕ* can be expressed as^31^:

3 $$\Phi (\lambda ,r)=\frac{P}{4\pi rD}\mathit{exp}(-{\mu }_{eff}r)$$

where *D* = *1/µ′*_*s*_ and P is the source power. Figure 4 shows *µ*_*eff*_ and *ϕ* as a function of wavelength for four increasing distances *r* from the injection point, and assuming an input power of 1 W. The average value of the optical properties of the fresh tissue were used to calculate the fluence rate (i.e., the filled squares and circles in Fig. 2). The spectral regions between 800–900 nm and 1050–1100 nm are substantially equivalent and offer the lowest tissue attenuation (in the 800–900 nm range the minimum value of *µ*_*eff*_ is 2.44 cm^−1^ @ 825 nm, whilst in 1050–1100 nm interval the minimum *µ*_*eff*_ is 2.49 cm^−1^ @ 1080 nm). Conversely, below 760 nm, the combination of scattering and increased absorption due to hemoglobin makes light penetration less effective. Moreover, a broad peak in *µ*_*eff*_ is observable at 980 nm owing to the water content. In the two low-attenuation ranges, the fluence rate is damped by around 2 decades every cm of distance from the source.

## Discussion

In summary, we report here for the first time a systematic overview of the broadband (600–1100 nm) optical properties of healthy porcine pancreatic tissue ex vivo.

Despite the arising need for novel diagnostic and therapeutic protocols for pancreas treatment, and the proven efficacy of optical therapies, such as LA^13^, only a restricted number of studies investigated the optical properties of pancreatic tissue. This represents a crucial aspect, as the optical properties are tissue specific and enable the complete understanding of the light-tissue interaction for both diagnosis and therapy. In particular, for the therapeutic purposes of LA, the implementation of pancreas-specific predictive tools would allow calculating the amount of heat generated by the pancreas-laser interaction and guiding the clinician in the selection of the best procedural settings.

The few works available in literature mainly focused on specific wavelengths or on a wavelength range that does not fully lie in the near-infrared optical therapeutic window (650–1350 nm). A study employing a double-integrating-sphere and an inverse Monte Carlo analysis algorithm reports the optical properties of ex vivo neuroendocrine pancreas tumor at 1064 nm (0.9 cm^−1^ in *µ*_*a*_ and 23 cm^−1^ in *µ′*_*s*_)^32^. The results obtained in the current study at 1065 nm for comparison are 0.17 cm^−1^ in *µ*_*a*_ and 12.6 cm^−1^ in *µ′*_*s*_.

Diffuse reflectance and fluorescence measurements were performed on both ex vivo and in vivo (pilot) human pancreatic tissues in the 400–700 nm range^33–35^. The studies show considerable promise in the use of optical techniques to distinguish normal pancreatic tissue from pathological ones (pancreatitis and adenocarcinoma). Another ex vivo study employed the use of inverse spectroscopic optical coherence tomography (ISOCT) in the wavelength range of 650–800 nm on duodenum biopsies from patients with and without pancreatic cancer^36^. The reported reduced scattering value as a function of depth varies between 20 and 40 cm^−1^ for both populations.

Spatially gated light scattering spectroscopy was used to distinguish in vivo, benign from malignant pancreatic cysts^37^. The diagnostic algorithm used in this study assumed a constant value of 30 cm^−1^ for the reduced scattering coefficient (our result is 20.4 ± 7.7 cm^−1^ @ 650 nm) and a negligible influence of absorption coefficient in the wavelength range of 600–800 nm. These values were assumed to simulate the contribution of backscattering and multiple scattering components for different source-detector separations, and to calibrate the probe using a tissue-equivalent phantom. Thus, the adoption of broadband spectral values derived from pancreatic tissues, as in our study, could be helpful in both procedures, i.e., to improve the measurement accuracy and reduce potential errors caused by incorrect assumptions.

Likewise, a feasibility study demonstrated the use of single fiber reflectance spectroscopy in vivo for optical guidance during endoscopic fine needle aspirations of pancreatic masses^38^. Optical properties (derived from the reflectance data and not reported in the study) were used to estimate blood volume, oxygen saturation, and bilirubin concentration with significant differences between the values for benign and malignant tissues.

In the present study, we performed the optical characterization of swine pancreatic tissues, starting from the assessment of the influence of different experimental factors on the measurement. In particular, the intra-sample variation, the compression testing, the repositioning error, and the measurement stability have been analyzed on fresh samples. Whereas *µ*_a_ and *µ*_s_′ spectra exhibit similar values in the mentioned testing conditions, we observed that the intra-sample variation has a significant effect only on *µ*_s_′ (median deviation ~ 11%). Concerning the optical properties of fresh samples, in average, *µ*_s_′ of fresh pancreas ranges from 12.1 cm^−1^ @ 1100 nm to 21.1 cm^−1^ @ 640 nm. The absorption coefficient *µ*_a_ of the pancreas ranges from 0.12 cm^−1^ @ 805 nm to 0.74 cm^−1^ @ 605 nm, with a peak of 0.42 cm^−1^ @ 980 nm (due to the water content). The general trends agree with *µ*_a_ and *µ*_s_′ values measured in ex vivo porcine brain and bone^26^. The attained values of *µ*_a_ and *µ*_s_′ can be included in mathematical frameworks for modeling the laser-pancreatic tissue interaction, light propagation, and subsequent photothermal effect at different wavelengths of the therapeutic window.

As previously reported for the liver^39,40^ the optical properties of the swine pancreas could reasonably approximate the properties of the human organ, with a tolerable difference which is compatible with the intra-sample variability. Indeed, Pifferi et al*.* have shown that a large intra-subject variability is present in human tissues, such as breast, resulting into a reduced scattering coefficient variability up to 1.6 times^41^. This intra-subject variability can affect the spatial heat conversion and temperature distribution and has to be taken into account when planning light dosimetry^42^.

Regarding the optical properties of frozen-thawed samples, we observed a threefold reduction of average values of *µ′*_*s*_ compared to the fresh tissues, whilst the values of *µ*_*a*_ were similar to fresh pancreas. The marked difference in *µ′*_*s*_ could be ascribed to the formation of ice within the examined tissues. Indeed, the intra- and extracellular ice formation occurring during freezing causes micro-changes of the tissue structure. The mechanical stress on the cell structures provoked by the ice crystals is at the origin of the cell damage. This mechanical phenomenon may lead to the homogenization of the tissue and, as a consequence, to the reduction of the scattering^43^.

These results suggest that freezing temperature, time, and method of tissue preservation must be carefully chosen for experiments relying on the accuracy of the optical response of the tissue, as the freezing procedure may lead to the variation of the optical properties of the specimen. As far as it concerns the evaluation of the tissue spectral attenuation and the measurement of *µ*_*eff*_ as a function of wavelength, we observed that the spectral bands between 800–900 nm and 1050–1100 nm provide the lowest tissue attenuation (*µ*_*eff*_ ranging from 2.4–2.5 to 2.7 cm^−1^) in the considered optical region, similarly to other tissues, such as brain^26^. Hence, wavelengths lying in these ranges can be employed in applications requiring higher light penetration within pancreatic tissue, whereas wavelengths in the 600–760 nm interval or close to the water peak at 980 nm are characterized by higher tissue attenuation (e.g., *µ*_*eff*_ = 4.14 cm^−1^ @ 980 nm). The results are in line with the widespread use of specific wavelengths such as: (1) 1064 nm, i.e., one of the most utilized wavelengths for LA of focal malignancies^10,13^, (2) 800–808 nm, laser wavelengths often employed in studies concerning nanoparticle-mediated photothermal therapies and requiring high penetration depth^44^, (3) 975–980 nm wavelengths, utilized for laser-assisted thermotherapies^45^ and typically associated with more elevated heating kinetics in biological media^46–48^ due to higher absorption. Hence, the present work offers an experimental validation from the optical point of view of the utilization of these wavelengths on pancreatic tissue and may provide indications also on other usable wavelengths toward the most suitable wavelength selection according to the specific therapeutic application.

The current study is performed on ex vivo tissue samples and more importantly on disease-free tissue. Both these features present a limitation to this study which we aim to overcome in upcoming studies. Ex vivo tissue could, over extended periods of time, lose moisture and blood content^49^. This is reflected as a reduction in the absolute value of the absorption coefficient. However, we have monitored the optical properties of the pancreas during 1 h time at room temperature, and no significant optical changes of the tissue were measured. We have observed that both absorption and scattering coefficients at 800, 900 and 1060 nm are rather stable over the test duration. This evidence suggests that there was no degradation of the tissue in the considered time span. These results are valid for the specific experimental conditions, and suggest that, in the future, the optical properties stability should be monitored for a longer time, in order to have a complete ex vivo characterization of the long-term degradation of hemoglobin on ex vivo samples. Conversely, in in vivo measurements, the organ will be continuously perfused and maintained in physiological conditions. Nevertheless, the oxygenation status of hemoglobin in vivo can be different from the ex vivo situation depending also on the physiological or pathological conditions.

The highly heterogeneous nature of the tissue under consideration could be another obvious drawback of the study. However, to take this into account we performed spatially separated measurements across different samples, and we did not observe major variations, as if the heterogeneous structure at a finer level was not affecting the macroscopic optical properties averaged over cm^3^ volumes. Finally, the sample exhibits strong absorption characteristics across the spectral region considered for this study. This coupled with the small thickness of the sample (< 1.5 cm) makes it difficult to model the photon migration even using highly accurate (MC) modeling^50^. The key limitation here is the finite temporal response of the detection instrument which is challenged for low thicknesses/high absorption causing the diffused transmitted photon temporal distribution to be almost indistinguishable from the system response. Yet, the convolution of the model with the system response can help in resolving even tiny differences in temporal broadening, as confirmed by the smooth scattering spectrum displayed in Fig. 2 that demonstrates the full system capabilities to disentangle absorption from scattering contributions.

Firstly, information on the optical characteristics may allow one to optimize the irradiation procedure by selecting the adequate laser specifications, e.g., laser wavelength, to obtain the required light penetration and absorption. Moreover, the attained tissue-specific optical coefficients are useful for implementing accurate predictive tools of the light-to-heat conversion for treatment planning^21,22^. These simulation-based models are particularly advantageous for estimating the light propagation and temperature distribution due to the photothermal effect. Accurate modeling can therefore support clinicians towards new treatment paradigms and the design of procedures specifically tailored to pancreatic tissue^23^. This is also crucial to determine the optimal procedural settings and the best LA strategy with the aim to improve the final clinical outcome.

## Conclusion

This work presents the optical characterization of ex vivo healthy porcine pancreas, in the wavelength range 600–1100 nm, by using TD-DOS. The absorption and reduced scattering coefficients (i.e., *µ*_a_ and *µ*_s_′) of the pancreas have been assessed in several experimental conditions, aiming at establishing a robust laboratory protocol. We have also estimated the optical properties of frozen-thawed pancreas samples, to investigate the effect of the freezing process on the optical response of the organ. The reduction of the scattering in frozen samples is ascribable to the tissue homogenization due to the cell damage caused by ice formation.

Lastly, the *µ*_*a*_ and *µ*_*s*_*′* spectra allowed us to calculate the fluence rate and the effective transport coefficient *μ*_*eff*_ for fresh pancreas and to identify the spectral regions associated with the lowest (800–900 nm and 1050–1100 nm) and highest (600–760 nm and close to 980 nm) tissue attenuation, along with spectral features due to tissue characteristics.

The data collected in this work provide for the first time a broadband characterization of the absorption and reduces scattering properties of pancreatic tissue, within a spectral range that is interesting for therapeutical aims, and biophotonic applications in general. The *µ*_*a*_ and *µ*_*s*_*′* coefficients reported here are useful for different purposes, such as the implementation of light-transport models to predict the outcome of laser-based therapies, and the formulation of pancreas-mimicking phantoms to be employed in the areas of diagnostics and therapy. Future investigations should consider the measurement of optical properties of human pancreas, in healthy and pathological conditions, in order to pave the way to patient-specific applications.

## Methods

### System set-up

**Figure 5:**
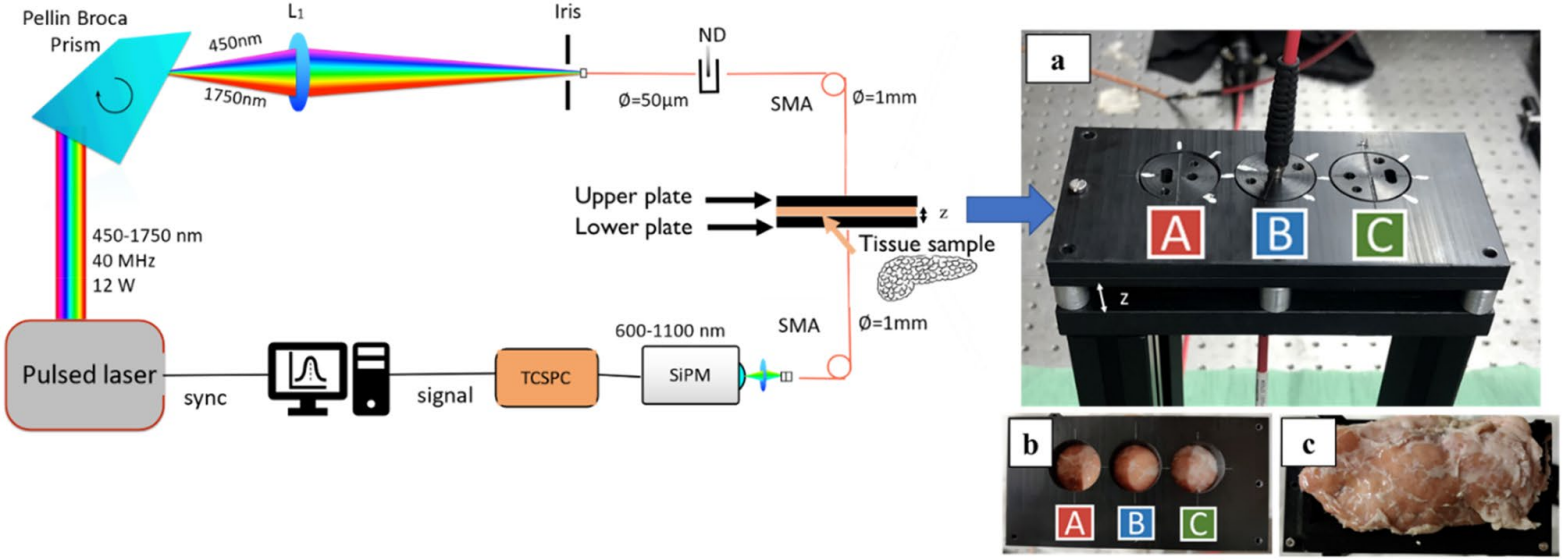
(Left) Schematic of the broadband diffuse optical spectrometer used to measure the ex vivo porcine pancreas optical properties. A pulsed broadband supercontinuum laser beam is spectrally dispersed by a Pellin Broca prism and cleaned by an iris before it enters the injection/source fiber. Temporal (ps) distribution of dispersed photons transmitted through the sample is detected by a Silicon Photomultiplier (SiPM) detector and resolved by a Time Correlated Single Photon Counting (TCSPC) board. (Right). (**a**) Image of PVC plates used to hold the source and the detection fibers and between which the sample is gently locked. A, B, and C indicate the three spatially separated locations chosen for the measurements; (**b**) top view of the upper plate before the injection of the source fiber; (**c**) image of one of the fresh porcine pancreatic samples positioned on the lower plate, which is used to hold the detection fiber.

The layout of the instrument used for this experiment is presented in Fig. 5. The system primarily comprises a pulsed supercontinuum laser emitting over a broad range of wavelengths (450–1750 nm) operated at a repetition rate of 40 MHz. This broadband pulsed light is dispersed using a Pellin Broca prism which allows for wavelength selection by rotation. The selected wavelength is then coupled into a 50 µm core optical fiber preceded by an iris. The iris and the small fiber core help in limiting the bandwidth of the selected wavelength to an average value of 5 nm in the wavelength range of interest (600–1100 nm). Light is then properly attenuated by a variable neutral density filter (ND) to cope with single-photon counting statistics and injected into the sample via a 1 mm core fiber. The sample is gently locked between two PVC plates with openings on either side that hold the source and the detection fibers, respectively. Light diffusively transmitted through the sample is collected on the other end of the sample using the detector fiber (1 mm core, step index) and focused onto a custom detection module based on a Silicon Photomultiplier (SiPM, S10362-11-050C, Hamamatsu, Japan) covering the whole 600–1100 nm range with reasonable responsivity^51^. The signal from the detector is then delivered to a Time Correlated Single Photon Counting (TCSPC) board which delivers the Distribution of Time Of Flight (DTOF) of detected photons. Further information regarding the instrumentation can be found elsewhere^52^. The instrument is completely automated and a typical measurement in the 600–1100 nm range at steps of 5 nm (100 wavelengths) takes around 2 min. The TD-DOS instrument was thoroughly validated following International Protocols for performance assessment of Diffuse Optics instruments, namely, the BIP, MEDPHOT, and NEUROPT protocols^53^. Also, a good agreement in the retrieval of the tabulated absorption spectrum of water in a diffusive aqueous phantom added with 1% Intralipid was demonstrated over the 600–1100 range.

### Data analysis

The standard analysis methodology used to recover the optical properties (*µ*_a_ and *µ′*_s_) from the measured DTOF curves is to fit them to an analytical solution of the Radiative Transport Equation under the Diffusion Approximation (DA)^54^. However, in thin samples with high absorption (as is the case in this study, thickness < 15 mm), the assumptions made under the DA are not satisfied, which could lead to inaccuracies in the estimation of the optical properties and a coupling between the two coefficients^55^. To avoid this issue, we employ a model based on fitting the DTOF curves to Monte Carlo (MC) simulations. The method is well established and involves generating a library of MC simulations at different reduced scattering coefficients at null absorption^56^. The effect of absorption is then accounted for by multiplying the factor $e^{{ - }{\mu_{a} vt}}$ in agreement with the radiative transfer equation. The simulated curve with a given set of optical properties is convoluted with the Instrument Response Function (IRF) and iteratively fit to the experimental DTOF curve using a Levenberg–Marquardt optimization algorithm to recover the optical properties. The IRF is acquired by placing the source and the detector fiber facing each other with a thin layer of Teflon in between. The DTOF curve was effectively used for the recovery of optical properties by choosing a fitting range between 80% of the peak value on the rising edge, down to 1% on the trailing edge. The time needed to fit a single DTOF (one point in the spectrum) was under 1 s.

### Experimental protocol

Porcine pancreases from 8 healthy pigs were extracted immediately after animal sacrifice from a local slaughterhouse. The organs were placed in a sealed bag, and an ice-water-cooled container was employed for transporting the organs to the laboratory assuring the absence of direct contact with ice or water. The samples were not treated with any anticoagulant prior to measurements. As described in detail in the following, 3 pancreases were employed for the sample compression, sample repositioning, and stability tests, respectively, whereas 5 pancreases were utilized for the measurement of the optical properties of the fresh and frozen-thawed organs. Moreover, the spatially separated measurements attained for one of these samples in the fresh state were also used to assess the intra-sample variation. Overall, our twofold analysis involved the assessment of measurement conditions and the actual measurement of the optical properties of the fresh and frozen-thawed samples.

#### Assessment of measurement conditions

Understanding the influence of different measurement conditions on the recovered optical properties could be of great interest as it gives an in-depth picture of the expected properties of the tissue used in different scenarios. Further, it provides a better appraisal of factors affecting the tissue properties. To this end, we studied the variation in the optical properties of the tissue for the following cases: spatially separated measurements on the same sample (i.e., intra-sample variation), compression testing, repositioning errors, and stability in the recovered optical properties over extended time periods.

**Figure 6:**
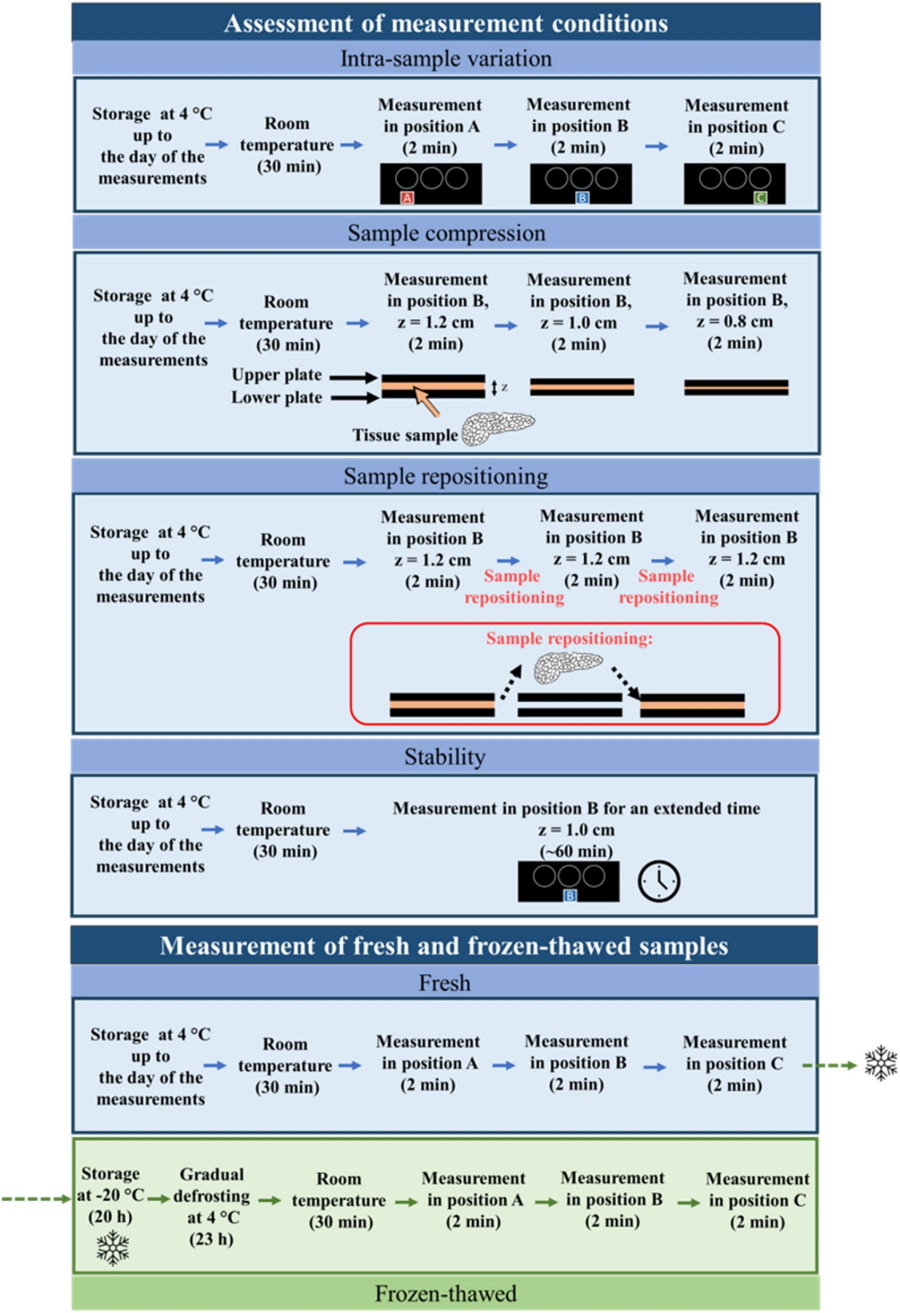
Schematic of the experimental protocol utilized for the assessment of the influence of the measurement conditions on the recovered optical properties and the estimation of the optical properties of the fresh and frozen-thawed samples.

The ex vivo pancreas tissues, upon arrival to the laboratory, were stored in the refrigerator at 4 °C until the beginning of the experiments. About 30 min before the starting of the measurements, the pancreatic tissue was removed from the refrigerator and kept at room temperature. Concerning the intra-sample variation investigations, three spatially separated locations were chosen for the measurement, i.e., positions A, B, and C (Fig. 6). For the compression testing, a sample with a thickness of 1.2 cm was measured first as it is and then pressed between the two PVC plates holding the sample to achieve uniform thicknesses of 1.0 cm and 0.8 cm and measured for both cases. To test for the variation in the optical property spectra with repositioning, measurements were performed at the same location on the same sample thrice (position B) but between each measurement, the sample was removed from in between the PVC plates and repositioned slightly. Finally, the stability in the recovered optical properties of the tissue was studied for extended periods (data shown for optical properties at 800, 900 and 1060 nm for a time span of 1 h). Except for the sample compression test, the samples were always measured in an uncompressed state. Overall, for the analysis of the influencing conditions, 10 measurements were performed.

#### Measurement of the optical properties of the fresh and frozen-thawed samples

For the fresh pancreas, 5 different samples procured from 5 different swines were considered and, on each, 3 spatially separated locations were chosen for the measurement. All the measurements concerning the fresh tissue samples were performed within 2 days from slaughter. For the evaluation of the optical properties of pancreatic tissue undergoing the freezing procedure and the estimation of the influence of the storage method on tissue characteristics, the five organs were placed in the freezer immediately after measurements on fresh tissues and stored at − 20 °C for 20 h. Afterward, the tissues were transferred to the refrigerator and kept at 4 °C for 23 h in order to gradually defrost. Following the same protocol utilized for fresh tissue samples, 30 min prior to measurements the defrosted pancreatic tissue was removed from the refrigerator and kept ​at room temperature. The tissue samples temperature at the beginning of the measurement was ~ 20 °C (monitored by a type K thermocouple). The measurements were performed without applying any compression to the samples (the average sample thickness was between 1.1 and 1.2 cm).

## Data Availability

The datasets generated during the current study are available in the Figshare repository, at the link: https://doi.org/10.6084/m9.figshare.19122221.
